# Asynchronous responses of soil microbial community and understory plant community to simulated nitrogen deposition in a subtropical forest

**DOI:** 10.1002/ece3.750

**Published:** 2013-09-16

**Authors:** Jianping Wu, Wenfei Liu, Houbao Fan, Guomin Huang, Songze Wan, Yinghong Yuan, Chunfeng Ji

**Affiliations:** 1 Institute of Ecology & Environmental Science, Nanchang Institute of TechnologyNanchang, 330099, China; 2 Hawkesbury Institute for the Environment, University of Western SydneyRichmond, NSW, 2753, Australia; 3 Key Laboratory of Vegetation Restoration & Management of Degraded Ecosystems, South China Botanical Garden, Chinese Academy of SciencesGuangzhou, 510650, China; 4 Department of Horticulture & Art, Jiangxi Agricultural UniversityNanchang, 330045, China

**Keywords:** Chinese fir, nitrogen deposition, plant diversity, PLFAs, soil microorganisms, South China

## Abstract

Atmospheric nitrogen (N) deposition greatly affects ecosystem processes and properties. However, few studies have simultaneously examined the responses of both the above- and belowground communities to N deposition. Here, we investigated the effects of 8 years of simulated N deposition on soil microbial communities and plant diversity in a subtropical forest. The quantities of experimental N added (g of N m^−2^ year^−1^) and treatment codes were 0 (N0, control), 6 (N1), 12 (N2), and 24 (N3). Phospholipid fatty acids (PLFAs) analysis was used to characterize the soil microbial community while plant diversity and coverage were determined in the permanent field plots. Microbial abundance was reduced by the N3 treatment, and plant species richness and coverage were reduced by both N2 and N3 treatments. Declines in plant species richness were associated with decreased abundance of arbuscular mycorrhizal fungi, increased bacterial stress index, and reduced soil pH. The plasticity of soil microbial community would be more related to the different responses among treatments when compared with plant community. These results indicate that long-term N deposition has greater effects on the understory plant community than on the soil microbial community and different conservation strategies should be considered.

## Introduction

Ecologists are increasingly recognizing that above- and belowground communities are tightly linked and that both communities greatly affect ecosystem processes and properties (Wardle et al. 2004; Bardgett et al. 2005). For example, plants influence soil biota by providing substrates in the form of litter and root exudates (Zak et al. 2003; Wardle et al. 2006), while decomposers regulate plant growth and community composition by mineralizing nutrients (Van Der Heijden et al. 2008). The feedbacks between above- and belowground communities are different among ecosystems and are affected by environmental factors (Bever 2003; Bardgett and Wardle 2010). Little is known, however, about the simultaneous responses of above- and belowground communities to changes in the global environment including changes in nitrogen (N) deposition (Suding et al. 2008; Bardgett and Wardle 2010).

Anthropogenic activities, such as the burning of fossil fuels, the application of artificial fertilizers, and the cultivation of N-fixing legumes, have altered the global N cycle (Vitousek et al. 1997; Galloway et al. 2004, 2008). Over the past century, atmospheric deposition of reactive N (mainly, nitrogen oxide and ammonia) has increased three- to fivefold (IPCC 2007), and N deposition is expected to further increase in many areas of the world (Galloway et al. 2004). N deposition could influence above- and belowground communities by altering soil chemical properties, plant community composition, and litter inputs (Bååth and Anderson 2003; Allison et al. 2007; Manning et al. 2008). Because N loading increases the quantity and quality of litter input and thus promotes decomposer abundance and activity (Manning et al. 2008; Cusack et al. 2011), N deposition can directly affect plant growth and soil communities (Manning et al. 2006). N deposition can also affect plant community composition by enhancing competitive release of N-limited species (Gilliam 2006; Bobbink et al. 2010). However, more research is needed on the responses of plants versus microorganisms to changing environments (Suding et al. 2008) because the mechanisms underlying the responses and interactions remain unknown. This is especially true for tropical and subtropical forest ecosystems, which not only harbor great biodiversity (Bobbink et al. 2010; Janssens et al. 2010) but which also have been confronted with increasing N deposition (Lamb et al. 2005; Galloway et al. 2008).

Substantial research has documented that increases in atmospheric N deposition could strongly affect plant community composition (Clark and Tilman 2008; Duprè et al. 2010; Maskell et al. 2010). Although N enrichment could increase plant growth by eliminating N deficiency (Vitousek and Howarth 1991; Lebauer and Treseder 2008), plant growth might not always increase in response to N addition because excess N may not be invested in the carboxylation step of photosynthesis (Bauer et al. 2004). In addition, plant responses to N supply depend on plant functional type; when the N supply is low, for example, increases in biomass are greater for herbaceous than for woody species and for trees than for shrubs (Xia and Wan 2008).

N deposition could also affect microorganisms in different ways in different ecosystems (Allison et al. 2007; Keeler et al. 2009). Long-term experiments in temperate forests often showed that soil microbial biomass was negatively correlated with N input (Wallenstein et al. 2006; Treseder 2008). In a mature tropical forest, Mo et al. (2008b) also reported that high nitrogen deposition significantly reduced soil microbial biomass after a 3-year treatment. However, results are yet to be consistent – there was one study found that large amounts of N fertilizer had no effect on soil microbial characteristics (Keeler et al. 2009) – which indicates that more detailed studies should be conducted on this topic.

Recent research in tropical ecosystems showed that N deposition affected the soil microbial community (Cusack et al. 2011), understory plant diversity (Lu et al. 2010), N cycling (Fang et al. 2011), and ecosystem carbon dynamics (Mo et al. 2008b). To our knowledge, however, no study has simultaneously measured the responses of plant and soil microbial communities to N deposition in subtropical regions.

This study concerns the effect of N on plant and soil microbial communities in Chinese fir plantations. Chinese fir (*Cunninghamia lanceolata*) is one of the most important reforestation species in subtropical China (Sheng et al. 2010). By 2009, Chinese fir plantations occupied approximately 1.12 × 10^7^ ha and represented the third largest reforested-plantation type in China (China Forestry Administration 2010). Although a recent report indicated that Chinese fir is sensitive to N deposition (Wei et al. 2012), it remains unclear how plant and soil microbial communities in Chinese fir plantations would respond to N deposition. The current report describes a long-term field experiment that included 8 years of continuous N application in a 20-year-old Chinese fir plantation. The objective was to test the effects of N deposition on both plant and soil microbial communities in the plantation.

## Materials and Methods

### Study site

The experiment was conducted at the Guanzhuang National Forestry Farm (117°43′E, 26°30′N), Sanming City, Fujian Province, South China. The area has a typical subtropical monsoon climate, with a mean annual precipitation of 1606–1650 mm and a mean annual temperature of 18.8–19.6°C. The soil is classified as acrisol. The Chinese fir plantation in this study was established in 1992 on hilly land with uniform site characteristics. Our experiment was initiated in December 2003 when the Chinese fir plantation was 12 years old. The plantation occupied 5173 ha. Fir saplings were planted with a spacing of 3 m × 2 m, resulting in about 1660 trees per ha. At the beginning of the experiment, the average tree height was 12 m, the mean diameter at breast height (DBH, 1.3 m) was 16.1 cm, mean soil organic carbon (SOC) was 18.39 g/kg, mean soil bulk density was 1.06 g/cm^3^, and mean soil pH was 4.68 (Fan et al. 2008).

### Experimental design

In a 6-ha section of the plantation, we established 12 experimental plots, each measuring 20 m × 20 m. Each plot was treated with one of four levels of N. The treatment codes and levels were N0 (control, 0 g N m^−2^ year^−1^), N1 (6 g N m^−2^ year^−1^), N2 (12 g N m^−2^ year^−1^), and N3 (24 g N m^−2^ year^−1^). Each treatment was represented by three replicate plots, which were randomly arranged. For treatment of a plot, the required quantity of CO(NH_2_)_2_ was dissolved in 20 L of water, and the solution was sprayed onto the soil surface plot. Control plots were treated with 20 L of water without CO(NH_2_)_2_. Plots were treated one time each month starting in January 2004 and continuing until sampling in 2011 (see next section).

### Sampling and analyses

Soils were sampled in September 2011 as the biomass of understory plants in southern China is usually highest in autumn. Three soil cores (3 cm diameter) were collected at depths of 0–20 cm, and three were collected at 20–40 cm; the cores were collected from the higher, middle, and lower part of each plot to account for any heterogeneity resulting from position on the slope. Plant litter was removed from the soil surface before the cores were taken. The three cores were combined to form one composite soil sample per depth per plot. Fresh soils were passed through a 2-mm sieve, and remaining roots and stones were removed by hand. Soil samples were divided in half; one half was used for determination of soil physicochemical characteristics, and the other half was used for phospholipid fatty acid (PLFA) analysis. Illuminance (an indication of fir canopy cover) was measured at 130 cm above the soil surface with a TES Digital Illuminance Meter (TES-1334A; Electrical Electronic Corp., Taibei, Taiwan) in September 2011.

For physicochemical analyses, soil samples were air dried, ground, and passed through a 0.25-cm sieve. SOC was measured by the Walkley–Black method. Total soil nitrogen was measured after micro-Kjeldahl digestion (Liu 1996). Soil pH was determined using a 1:2.5 ratio of soil mass to deionized water volume. Soil cation concentrations (including Mn, Ca, K, Al, Cu, Mg, and Zn) were determined by inductively coupled plasma mass spectrometry (ICP-MS; Agilent 7700, Santa Clara, CA) after acid digestion.

Soil microbial communities were examined by PLFAs analysis as described by Bossio and Scow (1998). Different PLFAs were considered to represent different groups of soil microorganisms. Bacterial PLFAs were represented by i15:0, a15:0, 15:0, i16:0, 16:1ω9, i17:0, a17:0, 17:1ω8, 17:0, cy17:0, 18:1ω7, and cy19:0. Fungal PLFAs were represented by the PLFAs 18:1ω9, 18:2ω6, and 18:3ω6 (Frostegård and Bååth 1996; Bossio and Scow 1998; Frostegård et al. 2011). The ratio of fungal PLFAs to bacterial PLFAs was used to estimate the ratio of fungal to bacterial biomass (fungi:bacteria [F:B]) in soils (Bardgett et al. 1996; Frostegård and Bååth 1996). The ratio of cy17:0 to 16:1ω7c was used as a bacterial stress index (BSI) (Grogan and Cronan 1997), an index that indicates microbial physiological status in response to environmental stress. Arbuscular mycorrhizal fungi (AM fungi), indicated by the PLFA 16:1ω5c (Olsson 1999; Van Diepen et al. 2010), were measured separately because these fungi can greatly affect plant carbon and nutrient balance. Taken together, all the PLFAs indicated by MIDI peak identification software (MIDI, Inc., Newark, DE) were considered to be the representatives of soil microbial communities.

For understory plant community analysis, we established one 5 m × 5 m subplot in each plot. All plants taller than 5 cm were recorded. The number of species and families, plant coverage, and plant abundance were evaluated. Understory plants were divided into four functional groups: tree seedlings, shrubs, vines, and herbaceous plants.

### Statistical analyses

One-way analysis of variance (ANOVA) was used to determine the effect of treatment on the plant and soil microbial communities. Two-way ANOVAs were used to determine effects of treatment, soil depth, and their interactions on soil microbial communities and soil physicochemical characteristics. For both kinds of ANOVAs, the levels of N were used as class variables. Regression analysis was used to test relationships between environmental factors and the plant community. These statistical analyses were carried out with SPSS 15 (SPSS, Inc., Chicago, IL). Significant was set at the 0.05 level.

Redundancy analysis (RDA) was used to determine which environmental factors were related to the composition of the soil microbial community. The most discriminating environmental factors were selected by the “forward selection” procedure of the program, and forward selection was based on Monte Carlo permutation (*n* = 499). Statistical significance tests for RDA were run using CANOCO software for Windows 4.5 (Ithaca, NY).

## Results

### Soil properties

**Figure 1:**
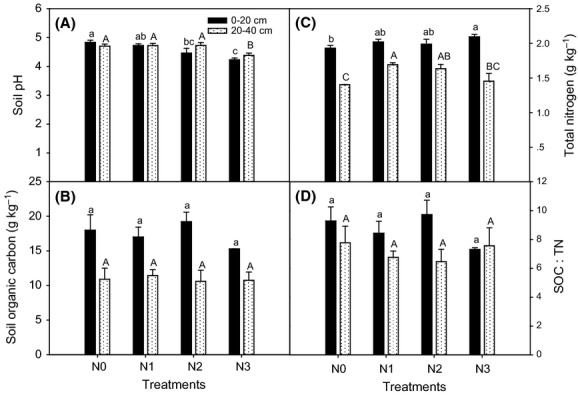
Soil pH (A), soil organic carbon (SOC) content (B), total soil nitrogen (TN) content (C), and the ratio of SOC to TN (D) as affected by N-deposition treatment and soil depth. Values are means ± SE, *n* = 3. Within each soil depth, values with a different letter are significantly different (*P* < 0.05). N0, N1, N2, and N3 refer to addition of 0, 6, 12, and 24 g of N m^−2^ year^−1^, respectively. The statistical effect of soil depth is indicated in Table 1.

After 8 years of simulated N deposition, soil pH at 0–20 cm depth was significantly lower in N2 and N3 plots than in N0 and N1 plots (Fig. 1A). The values of soil pH were decreased from 4.84 in N0 treatment to 4.24 in N3 treatment. Soil pH at 20–40 cm layer was significantly lower in N3 plots than in the other plots (Fig. 1A). The values of soil pH were decreased from 4.70 in N0 treatment to 4.39 in N3 treatment. Concentrations in the upper layer were not significantly different with the lower layer (Table 1; Fig. 1A). Soil pH was not significantly affected by depth or by the interaction between treatment and depth (Table 1). SOC content and the ratio of SOC to total soil nitrogen (TN) did not significantly differ among treatments at either depth (Fig. 1B and D), but was much greater at 0–20 cm depth than at 20–40 cm depth (Fig. 1B and D; Table 1). TN at 0–20 cm depth was significantly lower in N0 plots than in N3 plots (Fig. 1B). Concentrations of TN in different soil layers across the treatments were substantially different (Table 1; Fig. 1B). Except for Mn, concentrations of base cations did not significantly differ among treatments (Table S1).

**Table 1:** Effects of nitrogen addition (Treatment), soil depth (Depth), and their interactions on soil variables as indicated by two-way ANOVA statistics

Soil variable	Treatment		Depth		Treatment × Depth	
		
	*F*	*P*	*F*	*P*	*F*	*P*
Total PLFAs	3.69	**0.03**	44.01	**<0.001**	1.65	0.22
Bacterial PLFAs	4.59	**0.02**	54.09	**<0.001**	1.66	0.22
Fungal PLFAs	2.36	0.11	31.39	**<0.001**	1.38	0.29
F:B	0.64	0.60	0.18	0.68	0.99	0.42
AM fungal PLFAs	10.95	**<0.001**	61.01	**<0.001**	3.11	0.06
BSI	15.21	**<0.001**	12.01	**0.003**	0.15	0.93
SOC	0.64	0.60	41.04	**<0.001**	0.78	0.52
pH	10.44	**<0.001**	1.04	0.32	1.86	0.18
TN	3.85	**0.03**	127.2	**<0.001**	3.18	0.053
SOC:TN	0.60	0.62	5.91	**0.03**	1.26	0.32

ANOVA, analysis of variance; PLFAs, phospholipid fatty acids; AM, arbuscular mycorrhizal; BSI, bacterial stress index; SOC, soil organic carbon; TN, total soil nitrogen.

Values in bold are statistically significantly different at *P* < 0.05.

**Figure 2:**
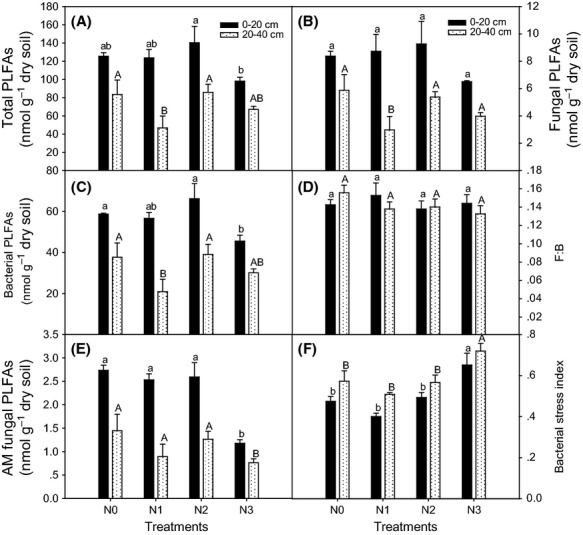
Soil microbial phospholipid fatty acids (PLFAs) as affected by N-deposition treatment and soil depth. Values are means ± SE, *n* = 3. Within each soil depth, values with a different letter are significantly different (*P* < 0.05). See Figure 1 for abbreviations. The statistical effects of soil depth are indicated in Table 1.

According to the two-way ANOVAs (Table 1), treatment and depth significantly affected total PLFAs (Fig. 2A), bacterial PLFAs (Fig. 2C), AM fungal PLFAs (Fig. 2E), and the BSI (Fig. 2F). Fungal PLFAs and the F:B ratio were unaffected by nitrogen deposition treatments in the 0–20 soil depth (Table 1; Fig. 2B and D). Bacterial and fungal PLFAs at 20–40 cm depth were significantly reduced by N1 treatment. Conversely, the BSI was higher in the N3 plots than in the other plots. The interactions between treatment and depth were not significant for any component of the microbial community (Table 1).

**Figure 3:**
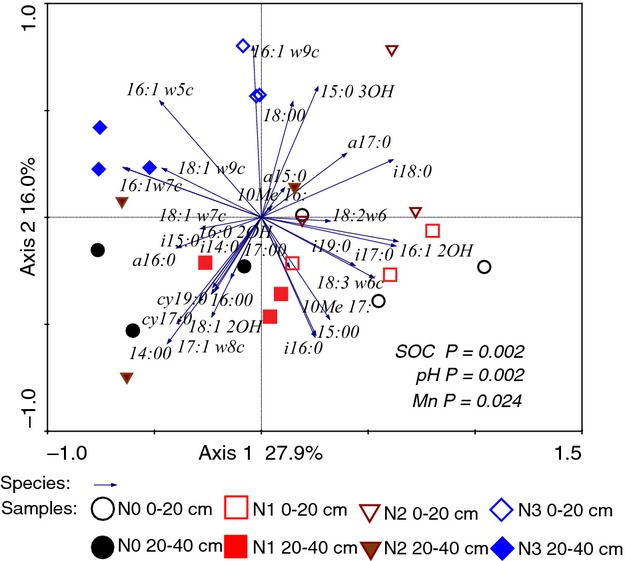
Redundancy analysis of soil microbial phospholipid fatty acids (PLFAs) as related to soil depth and N-deposition treatment. The ordination diagram presents species scores and treatment scores. Environmental factors shown in the diagram explain a significant portion of the soil microbial community data. See Figure 1 for abbreviations.

Ordination diagram revealed significant changes in the soil microbial community in response to soil depth (the first axis) and N deposition (the second axis) (Fig. 3). RDA indicated that the composition of soil microbial community was substantially related to SOC (*P* = 0.002), soil pH (*P* = 0.002), and Mn (*P* = 0.024); all the environmental data explained 63.2% of the variance, with axis 1 representing 27.9% and axis 2 another 16.0% of the variability (Fig. 3).

### Plant community

**Figure 4:**
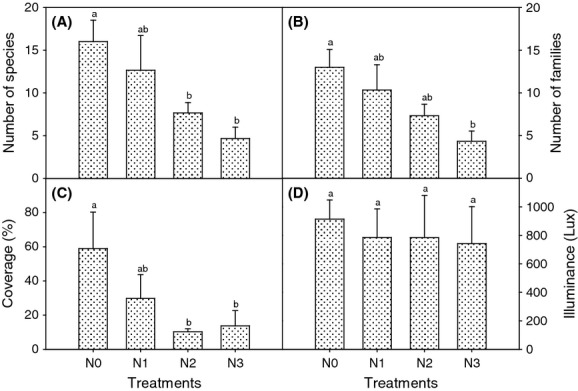
Effects of N deposition on plant richness (species and families), plant cover, and illuminance under the tree canopy. Values are means ± SE, *n* = 3. Within each panel, values with a different letter are significantly different (*P* < 0.05). See Figure 1 for abbreviations.

The number of species and plant coverage were significantly lower in N2 and N3 plots than in N1 and control plots (Fig. 4A and C). The number of families declined with N addition and was significantly lower in the N3 than in the other plots (Fig. 4B). As indicated by illuminance, canopy closure did not differ among treatments (Fig. 4D). Among all treatments, plant cover and richness were generally higher for tree seedlings than for the other functional groups (Tables 3). N addition tended to reduce the percentage of cover of vines and especially of tree seedlings and herbaceous plants and species richness but not of shrubs (Table 2). Similarly, N addition reduced the species richness of tree seedlings, vines, and herbaceous plants but not of shrubs (Table 3).

**Table 2:** Effects of nitrogen addition on plant cover (%) of different functional groups

Functional group	Treatment			

	N0	N1	N2	N3
Tree seedlings	46.54 ± 20.84a	17.72 ± 10.96ab	4.77 ± 1.14bc	0.72 ± 0.68c
Shrubs	4.81 ± 3.35a	5.12 ± 3.49a	1.41 ± 1.03a	10.46 ± 10.16a
Vines	6.67 ± 2.97a	3.66 ± 1.85a	0.87 ± 0.39b	2.51 ± 2.46a
Herbaceous plants	6.80 ± 2.29a	5.48 ± 5.41a	3.22 ± 3.22a	0.01 ± 0.01b

Values are means ± SE, *n* = 3. N0, N1, N2, and N3 refer to the addition of 0, 6, 12, and 24 g of N m^−2^ year^−1^, respectively. Values in a row followed by different letters are significantly different (*P* ≤ 0.05).

**Table 3:** Effects of nitrogen addition on species richness of different functional groups of plants

Functional group	Treatment			

	N0	N1	N2	N3
Tree seedlings	6.67 ± 1.20a	4.67 ± 0.33ab	3.67 ± 0.33b	2.00 ± 0.58c
Shrubs	2.00 ± 0.00a	2.33 ± 0.88a	1.33 ± 0.33a	1.00 ± 0.58a
Vines	5.67 ± 1.76a	3.00 ± 1.53ab	1.33 ± 0.33b	1.67 ± 0.88b
Herbaceous plants	6.33 ± 2.40a	4.00 ± 3.51ab	1.33 ± 1.33b	0.33 ± 0.33b

Values are means ± SE, *n* = 3. N0, N1, N2, and N3 refer to the addition of 0, 6, 12, and 24 g of N m^−2^ year^−1^, respectively. Values in a row followed by different letters are significantly different (*P* ≤ 0.05).

**Figure 5:**
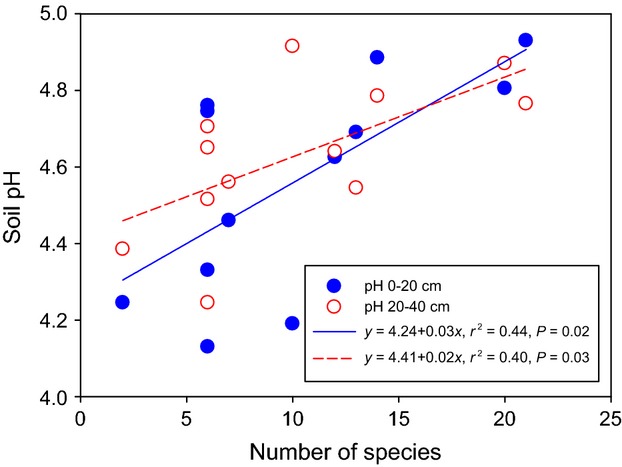
Relationships between soil pH at two depths (0–20 cm, solid circles with solid line; 20–40 cm, open circles with dash line) and number of understory plant species per plot as affected by N deposition. Each point is the mean of one replicate plot.

**Figure 6:**
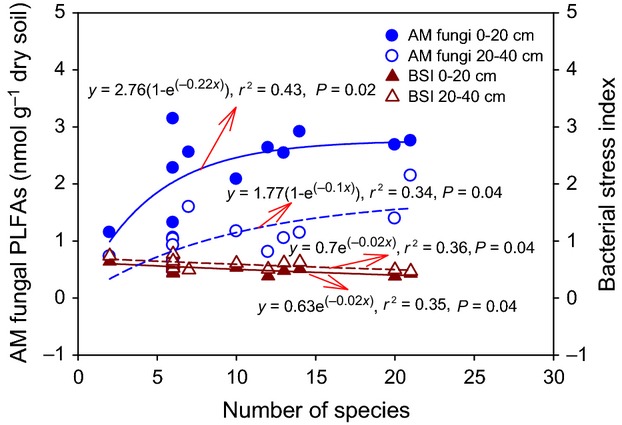
Relationships between the abundance of soil arbuscular mycorrhizal (AM) fungi at two depths (0–20 cm, solid circles with solid line; 20–40 cm, open circles with dash line) and bacterial stress index (BSI) at two depths (0–20 cm, solid triangle with solid line; 20–40 cm, open triangle with dash line) and number of understory plant species per plot. Each point is the mean of one replicate plot.

Regression analysis showed that species richness of understory plants was positively related to soil pH at both depths (Fig. 5). Regression analysis also showed that species richness of AM fungi at both depths was positively related to species richness of understory plants (Fig. 6). In contrast, BSI at both depths was negatively related with species richness of understory plants (Fig. 6).

## Discussion

### Linking responses of plants and soil microorganisms

In this 8-year study, soil microbial levels at 0–20 cm depth (as indicated by PLFA abundances) were only reduced by addition of N at the highest rate (24 g of N m^−2^ year^−1^ in the N3 plots) while plant diversity declined progressively with each increase in the rate of N addition, indicating that N deposition affected the plant community more than the microbial community in the long-term nitrogen addition. This was unexpected because a previous study had proposed that the soil microbial community would respond more quickly than the plant community to environmental change (Schmidt et al. 2007). Because soil microbial communities can acclimate to environment changes (Luo et al. 2001; Marshall et al. 2011), we postulate that the soil microbial community at 0–20 cm depth in plots treated with 6 or 12 g of N m^−2^ year^−1^ may have been altered in the short term, but then acclimated to N deposition so that, after 8 years of treatment, microbial communities were similar in plots treated with ≤12 g of N m^−2^ year^−1^. The response of the soil community to N addition, however, differed greatly with depth. Whereas only the highest rate of N addition reduced the abundance of bacterial PLFAs at 0–20 cm depth, only the lowest rate (6 g of N m^−2^ year^−1^) significantly reduced bacterial and fungal PLFAs at 20–40 cm depth. Explaining this effect of soil depth on the response of the soil microbial community to N addition will require additional research.

The highest rate of N addition reduced the diversity and cover of understory plants, and the reduction in plant diversity and cover was correlated with a reduction in AM fungal PLFAs and an increase in the BSI. The decrease in diversity and cover of understory plants may have resulted in a decrease in belowground carbon allocation, which would contribute to decreases in soil microbial diversity and the available nutrient. Soil microorganisms are usually carbon limited, and decreases in root exudates strongly influence the soil microbial community (Högberg et al. 2007). Furthermore, the relationships between AM fungi and plant roots are usually mutualistic symbiosis, which also support our results.

In a previous microcosm experiment, ecosystem responses to N deposition were dominated by changes in plant growth and in the soil community rather than changes in plant community composition (Manning et al. 2006). In a previous field experiment, in contrast, plant community composition was initially resilient but then was significantly altered by increasing N availability (Suding et al. 2008). Our results were consistent with those of the latter study in that the responses to N addition were stronger for plant community composition than for soil microbial biomass (as indicated by total PLFAs) or bacteria:fungi (as indicated by the ratio of bacterial and fungal PLFAs).

### Soil responses

Although the effect was not statistically significant, soil microbial biomass as estimated by total PLFAs was 21.8% lower in the N3 plots (which had the highest level of N addition) than in the control plots. A previous investigation at the same study site found that litter decomposition was inhibited by the N3 treatment (Fan et al. 2008), indicating that the high level of N addition suppressed soil microbial activities. Soil pH in this study was also significantly reduced in the N3 plots, and soil pH is strongly correlated with soil microbial biomass (Bååth and Anderson 2003). A similar trend was also reported for a subtropical forest in that a high level of N deposition, rather than low or middle levels, decreased soil microbial biomass (Mo et al. 2008b). Other studies reported reductions in soil microbial biomass of 15–50% as a consequence of N fertilization (Wallenstein et al. 2006; Treseder 2008; Liu and Greaver 2010).

Like total PLFAs, both fungal and bacterial PLFAs declined in the N3 plots (although the decline in fungal PLFAs was not statistically significant) at 0–20 cm depth. Short-term elevated N deposition may not significantly change fungal biomass (Waldrop et al. 2004), but fungal biomass can be reduced by long-term, elevated N deposition (Frey et al. 2004). Previous studies reported that bacterial biomass can be suppressed by N deposition (Compton et al. 2004; Frey et al. 2004). Because fungi and bacteria were similarly suppressed by N addition, the ratio of fungal to bacterial PLFAs changed very little. Still, we suspect that N deposition substantially changed the soil microbial community as revealed by RDA in our study. Compton et al. (2004) found that although N deposition did not change fungal and bacterial biomass, it did greatly affect other characteristics of the soil microbial community. In this study, N addition reduced AM fungi. We speculate that this finding can be at least partially explained by a decline in carbon allocation to AM fungi (Van Diepen et al. 2010) because most photosynthates would first favor the associated mycorrhizae in plant–microbe networks (Leake et al. 2004).

The SOC concentration in this study was not significantly changed by N addition, which is inconsistent with some other long-term experimental results in temperate and boreal forests (De Vries et al. 2006; Pregitzer et al. 2008). Increased carbon in soil is primarily caused by declines in organic matter decomposition (Pregitzer et al. 2008), and other decomposition studies indicate that N enrichment can stimulate soil carbon sequestration (Waldrop et al. 2004; Hobbie 2008). Conversely, a recent review suggested that carbon content in mineral soil was not altered by N addition (Liu and Greaver 2010), which is consistent with our results. The potential explanation is that activities of soil microbial decomposers were reduced by N addition, resulting in most of the litter carbon remaining on the surface soil layer instead of being mixed with the mineral soil by soil decomposers (Liu and Greaver 2010). This inference is supported by the finding that soil microbial biomass (as indicated by total PLFAs) and soil carbon concentrations were greater at 0–20 cm than at 20–40 cm depth and that the opposite was true for the BSI. Li et al. (2006) also reported that fertilization had only marginal effects on total SOC in a tropical forest.

### Plant responses

Plant species richness and coverage were reduced in N2 and N3 plots after 8 years of N addition. Other long-term studies of N deposition indicated that understory richness was significantly decreased by high doses of N in mature subtropical forests, but that the effects were much smaller in disturbed or rehabilitated forests (more than 50-year-old forests) (Lu et al. 2010, 2011). We suspect that these differences reflect differences in the properties of undisturbed, mature versus disturbed systems. Although the trees in this study were only 20 years old and the plantation system was far from mature, the response to N addition was more similar to that reported for mature than for immature forests. Because plant growth in many terrestrial ecosystems is limited by N availability (Vitousek and Howarth 1991), increased N availability can promote species that can compete for N and that can grow rapidly in the absence of N limitation (Bobbink et al. 2010). A field biodiversity survey across the United Kingdom indicated that the significant decline in forbs could be ascribed to the competitive exclusion by nitrophilous grasses (Stevens et al. 2006). Similar mechanisms have also been proposed in temperate and boreal forest ecosystems (Gilliam 2006).

The reduced plant species richness and cover in response to N addition were not caused by canopy closure because the canopy closure (as indicated by illuminance) and DBH of the fir trees did not differ among the treatments (Figs. S1, S2). The reductions in understory plant diversity and cover were related to declines in soil pH. A previous report from our study site suggested that N loading resulted in soil acidification (Shen et al. 2012). Excess N deposition can increase nitrification activity and increase N leaching, especially with the abundant precipitation in South China (Fang et al. 2011). Surface soil nitrogen just significantly increased in N3 treatment, but not for N1 and N2 treatment, which would be due to the increased leaching after nitrogen enrichment (Macdonald et al. 2002). Declines in soil pH after N loading and consequent decreases in plant survival rates were detected in a field experiment with two tropical tree seedlings in South China (Mo et al. 2008a). Other temporal and spatial evidence also indicate that low soil pH can greatly reduce plant diversity (Duprè et al. 2010; Maskell et al. 2010). It is notable that soil base cations (Ca, Mg, Al, K, etc.) were not reduced, which supports the chronic responses for forest ecosystems to N deposition (Waldrop et al. 2004).

One limitation of our study is that the dosages of N applied were larger than ambient deposition rates (Högberg et al. 2006). Meanwhile, the Chinese fir plantations would not absorb much nitrogen from the atmosphere. According to our previous investigation in the Chinese fir plantations, the amount of captured nitrogen was small (0.56–2.97 kg N ha^−2^ year^−1^ reported by Fan and Hong (2001)) compared with the nitrogen gradient in this study. The loss in the diversity of herbaceous plant species is greater with a long-term, low level of N deposition than with a short-term, high level of N deposition (Clark and Tilman 2008). Indeed, He and Barclay (2000) found that N fertilization with more than 200 kg N ha^−2^ year^−1^ had minimal effects on understory species growth and diversity.

In conclusion, after 8-year treatment, the results indicate that the aboveground plant community and the belowground microbial community differ in their responses to N deposition. Based on the long-term investigation, understory plants would be more sensitive than soil microorganisms to simulated N deposition, and therefore separate conservation strategies should be considered for these two components in future climatic scenarios.

## Supporting Information

**Figure S1.** Average diameter at breast height (DBH, 1.3 m) in March 2012 after different nitrogen treatments.

**Figure S2.** Microclimates (A: atmospheric temperature; B: atmospheric humidity) of the plantation under different nitrogen treatments. Atmospheric temperature and humidity were determined by TES Humidity/Temperature Meter (TES-1361, Electrical Electronic Corp).

**Table S1.** The mean values of soil ions in 0–20 cm and 20–40 cm layers after 8 years of nitrogen treatments. Statistical analysis only found that the Mn element showed significant changes among nitrogen treatments as indicated by different labels.
