# Supplementary material for: Asynchronous responses of soil microbial community and understory plant community to simulated nitrogen deposition in a subtropical forest
Source: Ecol Evol. 2013 Sep 16;3(11):3895–905. doi: 10.1002/ece3.750 (PMC3810882; doi:10.1002/ece3.750)
Supplement: Supplementary file 1 [file ece30003-3895-SD1.doc]

Table S1 The mean values of soil ions in 0-20 cm and 20-40 cm layers after 8 years of nitrogen treatments. Statistical analysis only found that the Mn element showed significant changes among nitrogen treatments as indicated by different labels.

| Horizon and ions | Treatments | | | | | | | |
| --- | --- | --- | --- | --- | --- | --- | --- | --- |
|  |  | N0 |  | N1 |  | N2 |  | N3 |
| 0-20 cm soil depth |  |  |  |  |  |  |  |  |
| Ca |  | 1.69±0.47 |  | 1.52±0.01 |  | 0.65±0.30 |  | 2.05±0.31 |
| Mg |  | 2.08±0.36 |  | 2.40±0.22 |  | 1.36±0.50 |  | 2.71±0.59 |
| Al |  | 81.27±24.16 |  | 79.53±3.34 |  | 57.00±21.84 |  | 110.86±19.93 |
| K |  | 5.14±0.78 |  | 7.43±0.04 |  | 5.10±1.98 |  | 9.16±1.38 |
| Mn |  | 0.45±0.07a |  | 0.38±0.01a |  | 0.18±0.07b |  | 0.36±0.07ab |
| Fe |  | 32.20±7.91 |  | 36.12±1.54 |  | 24.88±9.41 |  | 49.78±4.46 |
| Cu |  | 0.06±0.02 |  | 0.04±0.00 |  | 0.05±0.02 |  | 0.11±0.05 |
| Zn |  | 0.13±0.05 |  | 0.11±0.01 |  | 0.08±0.03 |  | 0.23±0.07 |
| 20-40 cm soil depth |  |  |  |  |  |  |  |  |
| Ca |  | 0.86±0.47 |  | 2.07±0.74 |  | 0.89±0.32 |  | 1.80±0.00 |
| Mg |  | 1.91±0.86 |  | 3.59±0.82 |  | 1.76±0.53 |  | 1.04±1.03 |
| Al |  | 65.36±28.74 |  | 142.07±43.24 |  | 60.89±18.49 |  | 71.21±0.00 |
| K |  | 8.58±4.06 |  | 11.45±4.66 |  | 5.07±1.08 |  | 6.77±0.00 |
| Mn |  | 0.23±0.08b |  | 0.77±0.03a |  | 0.30±0.10b |  | 0.11±0.11b |
| Fe |  | 29.74±13.73 |  | 57.33±15.45 |  | 30.23±10.09 |  | 37.10±0.00 |
| Cu |  | 0.07±0.03 |  | 0.06±0.02 |  | 0.05±0.02 |  | 0.03±0.03 |
| Zn |  | 0.10±0.05 |  | 0.21±0.07 |  | 0.11±0.04 |  | 0.11±0.11 |

Figure S1 Average diameter at breast height (DBH, 1.3 m) in March 2012 after different nitrogen treatments.

Figure S2 Microclimates (a: Atmospheric temperature; b: Atmospheric humidity) of the plantation under different nitrogen treatments. Atmospheric temperature and humidity were determined by TES Humidity/Temperature Meter (TES-1361, Electrical Electronic Corp., Taiwan).
